# The quinic acid derivative KZ-41 prevents glucose-induced caspase-3 activation in retinal endothelial cells through an IGF-1 receptor dependent mechanism

**DOI:** 10.1371/journal.pone.0180808

**Published:** 2017-08-10

**Authors:** Hui He, Rebecca L. Weir, Jordan J. Toutounchian, Jayaprakash Pagadala, Jena J. Steinle, Jerome Baudry, Duane D. Miller, Charles R. Yates

**Affiliations:** 1 Department of Pharmaceutical Sciences, UTHSC College of Pharmacy, Memphis, Tennessee, United States of America; 2 Biochemistry and Cellular and Molecular Biology, The University of Tennessee, Knoxville, Tennessee, United States of America; 3 Department of Anatomy, Wayne State University, Detroit, Michigan, United States of America; 4 UT/ORNL Center for Molecular Biophysics, Oak Ridge National Laboratory, Oak Ridge, Tennessee, United States of America; 5 Department of Ophthalmology, UTHSC College of Medicine, Memphis, Tennessee, United States of America; Faculty of Medicine & Health Science, UNITED ARAB EMIRATES

## Abstract

Retinal microaneurysms, an early disease manifestation of diabetic retinopathy, are associated with retinal endothelial cell (REC) death and macular edema. We previously demonstrated that a quinic acid (QA) analog, KZ-41, promoted REC survival by blunting stress-induced p38 MAPK activation. Herein, we sought to expand our understanding of the pro-survival signal transduction pathways actuated by KZ-41. Using human RECs exposed to high glucose (25 mM, 72 hours), we demonstrated that KZ-41 blocks caspase-3 activation by triggering phosphorylation of the PI3K regulatory subunit (p85; Tyr458) and its downstream target Akt (Ser473). Akt signal transduction was accompanied by autophosphorylation of the receptor tyrosine kinase, insulin growth factor-1 receptor (IGF-1R). IGF-1R knockdown using either the tyrosine kinase inhibitor AG1024 or silencing RNA abolished KZ-41’s pro-survival effect. Under high glucose stress, caspase-3 activation correlated with elevated ERK1/2 phosphorylation and decreased insulin receptor substrate-1 (IRS-1) levels. KZ-41 decreased ERK1/2 phosphorylation and reversed the glucose-dependent reduction in IRS-1. To gain insight into the mechanistic basis for IGF-1R activation by KZ-41, we used molecular modeling and docking simulations to explore a possible protein:ligand interaction between the IGF-1R kinase domain and KZ-41. Computational investigations suggest two possible KZ-41 binding sites within the kinase domain: a region with high homology to the insulin receptor contains one potential allosteric binding site, and another potential site on the other side of the kinase domain, near the hinge domain. These data, together with previous proof-of-concept efficacy studies demonstrating KZ-41 mitigates pathologic retinal neovascularization in the murine oxygen-induced retinopathy model, suggests that QA derivatives may offer therapeutic benefit in ischemic retinopathies.

## Introduction

Diabetic retinopathy (DR), the most frequently occurring microvascular complication of diabetes, is a leading cause of vision loss. Retinal microaneurysms, an early disease manifestation, are associated with retinal endothelial cell (REC) death, capillary dropout, and macular edema [1]. The resultant ischemia triggers hypoxia-induced factor-1 (HIF-1) driven VEGF, eNOS, and ET-1 expression, which are biomarkers of retinal neovascularization (RNV) [2]. Acellular capillary formation in response to hypoxia exacerbates vascular leakage thus propagating a cycle of ischemia and pathological RNV. A better understanding of the mechanisms contributing to glucose-induced REC death may provide novel targets for the development of treatments for DR.

Prolonged high glucose exposure inactivates Akt-dependent pro-survival signaling leading to reduced endothelial cell viability [3]. Overexpression of constitutively active Akt mutants rescues endothelial cells from glucose-induced apoptosis [4]. In macro- and microvessels of obese rats, insulin-stimulated tyrosine phosphorylation of both the insulin receptor beta (IR-β) subunit and insulin receptor substrates 1 and 2 (IRS-1 and IRS-2) is reduced [5]. Consequently, insulin-dependent IRS-1/2 recruitment of p85, a subunit of phosphatidylinositide 3-kinase (PI3K), and Akt activation are significantly reduced in isolated microvessels from obese rats compared to lean controls. Impaired insulin signaling, as evidenced by a reduction in IRS-1-dependent Akt activation, is evident in RECs exposed to high glucose [6].

Retinal Akt expression is reduced at eight and 12 weeks in streptozotocin-induced diabetic rats [7]. In the mouse retina, insulin growth factor-1 receptor (IGF-1R) and the less abundant insulin receptor (100-fold lower expression) are expressed in photoreceptors and endothelial cells [8]. Subcutaneous IGF-1 administration decreases retinal apoptosis in diabetic rats at 12 weeks as evidenced by a reduction in TUNEL-positive cells in the photoreceptor, inner nuclear, and ganglion cell layers [9]. IGF-1 triggers autophosphorylation of the IGF-1R kinase domain at tyrosine residues 1131, 1135, and 1136 followed by recruitment of specific docking intermediates (*e*.*g*., IRS-1), which links the IGF-1R to the PI3K/Akt signaling cascade [10]. In R28 cells, a neural cell line derived from the neonatal rat retina, IGF-1 inhibits caspase-3 activation and apoptosis via a PI3K/Akt-dependent mechanism [11]. In human RECs, IGF-1 required PI3K/Akt signal transduction to rescue cells from apoptosis secondary to high glucose or serum starvation. However, stress-induced proliferation required ERK activation, but was independent of PI3K/Akt signal transduction [12].

No FDA approved treatment exists for complications of DR, which include diabetic macular edema or RNV. However, anti-vascular endothelial growth factor (VEGF) monoclonal antibodies are commonly used off-label (*i*.*e*., non-FDA approved) as a treatment. Unfortunately, as many as 50% of patients treated with anti-VEGF agents fail to respond. Thus, our group is engaged in the identification of novel targets and therapeutic options designed to protect RECs from environmental stress. Toward this end, we have discovered a new class of orally bioavailable quinic acid (QA) analogs [13], which counteract p38 MAPK-dependent pro-apoptotic signaling in human RECs exposed to genotoxic stress including radiation and melphalan [14, 15].

Herein, we have expanded our understanding of the pro-survival mechanism of action of QA analogs using the *in vitro* model system of RECs exposed to high glucose. Specifically, a QA analog, KZ-41, reverses high glucose-induced caspase-3 activation in RECs by enhancing PI3K/Akt pro-survival signaling. Here we use computational approaches to propose a binding mechanism of KZ-41 in IGF-1R. Further, the IGF-receptor 1 (IGF-1R) appears indispensable to KZ-41’s mechanism of action since pharmacologic and genomic knockdown of IGF-R1 ablates KZ-41’s pro-survival activity. Though, its activity at the level of the IGF-1R differs from its endogenous ligand, IGF-1, with respect to ERK-mediated signaling [16].

## Materials and methods

### Reagents

**Fig 1:**
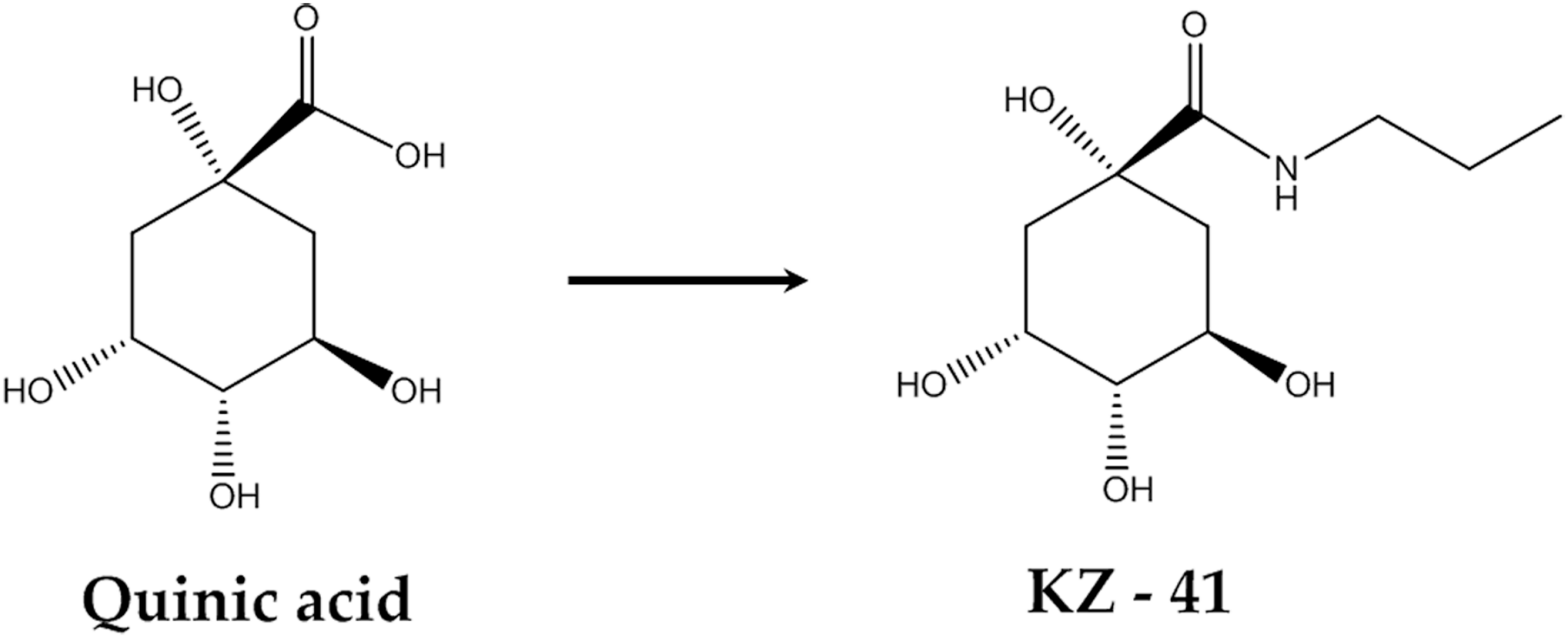
Quinic Acid and KZ-41 Structure. KZ-41 (molecular weight 233) represents a metabolically stable derivative of the natural product quinic acid (molecular weight 192).

Total IGF-1R, IRS-1, p85, ERK1/2 and Akt and phosphorylated (Tyr1135/1136) IGF-1R, (Tyr458) p85, (Thr202/Tyr204) ERK1/2, (Ser473) Akt, and GAPDH antibody (rabbit) primary antibodies were obtained from Cell Signaling (Danvers, MA). Secondary goat anti-rabbit IgG antibodies (IRDye 800CW) were purchased from LI-COR Biotechnology (Lincoln, NE). AG 1024, a specific IGF-1R phosphorylation inhibitor, was purchased from Selleck Chemicals (Houston, TX). IGF-1R siRNA was obtained from Cell Signaling (Danvers, MA). D-mannitol and glucose were purchased from Sigma (St. Louis, MO). KZ-41 (Fig 1) was synthesized in Dr. Duane Miller’s laboratory and verified to be >96% pure by nuclear magnetic resonance spectroscopy [17].

### Cell culture

Primary human retinal microvascular endothelial cells (RECs, Lot 181) were acquired from Cell Systems Corporation (CSC, Kirkland, WA). Only primary cells within passage six were used. Cells were routinely cultured in M131 medium containing microvascular growth supplements (Invitrogen, Carlsbad, California), gentamicin (10 μg/mL), and amphotericin B (0.25 μg/mL). Cells were then transferred to high (25 mM), normal (5 mM) glucose, or mannitol (25 mM) medium and cultured for three days. Prior to each experiment, cells were quiesced by incubating without growth supplements for 24 hours. KZ-41 (10 μM) was added and cell lysates were harvested after incubation for two hours unless otherwise indicated. In separate experiments, RECs were pre-incubated with the IGF-1R tyrosine kinase inhibitor AG1024 (10 μM) to investigate the role of IGF-1R autophosphorylation in the signal transduction of KZ-41.

### Caspase-3 activity

The PathScan cleaved caspase-3 (Asp175) sandwich enzyme-linked immunosorbent assay kit (Cell Signaling) was used to evaluate endogenous cleaved caspase-3 levels in REC lysates according to the manufacturer's instructions. For all ELISA analyses, equal protein amounts were loaded into each well, allowing for comparisons using optical density (O. D.).

### PTP1B activity

Protein tyrosine phosphatase 1B (PTP1B) activity was measured in a cell-free system using a colorimetric purified recombinant enzyme assay kit (EMD Millipore, Billerica, MA) in accordance with manufacturer’s protocol. Briefly, assay buffer (10 μL), KZ-41 (10 and 100 μM) or PTP1B inhibitor suramin (100 μM), was added to separate wells of a 96-well microtiter plate. Purified human recombinant PTP1B enzyme (5 μL) was added to each well to a final concentration of 2.5 ηg/mL. The reaction was started by adding the IR5 phosphate substrate (50 μL). After one hour of incubation at 30°C, the reaction was terminated using the Red reagent (25 μL). Color was allowed to develop for 30 minutes and absorbance was measured at 620 ηm using a microplate reader. Data represent mean ± SD, taken as a percentage of positive control, from triplicate experiments.

### siRNA transfections

After overnight serum starvation, RECs were transfected with IGF-1R siRNA (40 nM) or control (scrambled siRNA) (Cell Signaling) using lipofectamine RNAimax (Invitrogen) in high glucose (25 mM) medium for 72 hours followed by treatment with KZ-41 (10 μM). The transfection protocol was verified as optimal for knockdown of IGF-1R by Western blot analysis. Sham-transfected cells (without siRNA) served as an additional negative control.

### Western blotting analysis

Cellular proteins were analyzed by Western blot as previously described [14]. Briefly, REC lysates were collected in RIPA lysis buffer supplemented with protease/phosphatase inhibitor (1X) cocktail (Roche; Indianapolis, IN) and total protein was measured using the BCA assay (Pierce, Rockford, IL). Protein samples were loaded on NuPAGE 4–12% Bis-Tris gel (Invitrogen, Carlsbad, CA). Immunoblotting was performed with nitrocellulose membranes (Bio-Rad), blocked using Odyssey blocking buffer (LI-COR, Lincoln, NE), and incubated at 4°C with specific primary antibodies (1:1000) overnight. Cellular protein was normalized using GAPDH [1:10,000] (Cell Signaling). The secondary antibody (IRDye 800CW goat anti-rabbit) [1:10,000] was incubated in the dark at room temperature for 45 minutes. Dual-channel infrared scan and quantitation of immunoblots were conducted using the Odyssey Sa infrared imaging system with Image Studio (Ver. 3.1.4) (LI-COR).

### Statistical analysis

All data in the different experimental groups are expressed as mean ± S.D. and were obtained from at least three independent experiments. Analysis of variance (ANOVA) was used to assess the statistical significance of the differences between groups, followed by Duncan's multiple-range test or Student's t-test, where appropriate. A P value of < 0.05 was considered significant.

### Molecular modeling and docking calculations

All modeling and calculations were performed using the program MOE version 2016.08 (Chemical Computing Group Ltd., Montréal, Canada) and the force field Amber99 as implemented in MOE. A crystal structure of the kinase domain of IGF-1R exists with a co-crystallized inhibitor [18], and has been deposited in the PDB [19] (PDB ID: 3LW0). This structure includes a co-crystallized inhibitor, CCX (3-cyano-N-1H-indole-7-carboxamine)[20, 21]. However, the 1097–1105 and 1169–1171 domains of the protein do not have coordinates in the IGF-1R crystal structure. Ten homology models that include these 1097–1105 and 1169–1171 domains’ missing coordinates were built using MOE’s homology modeling facility, using the crystal structure of the IGF-1R kinase protein as a template, and keeping the CCX inhibitor in the models.

The three models (model 5, model 7, and model 4) with the smallest RMSD values compared to the crystal structure of the template were selected for further consideration. Amino acid residue pKa values were estimated at pH 7 and protonated accordingly using the Protonate3D facility in MOE. The resulting 3 models underwent a gradual energy minimization that occurred in three stages: i) atoms N12 of 3WL0.CCX1, N12 of 3LW0.A:CCX1287, and N34 of 3LW0.D:CCX1287 were kept fixed, as well as the alpha carbons of the protein backbone; ii) protein alpha carbons were unfixed; iii) all atoms were unfixed. Residues with alternate locations of atoms and atoms with fractional occupancies were assigned coordinates that correspond to the highest occupancy.

In each of the three models, i) the co-crystallized CCX inhibitor, ii) KZ-41, and iii) ATP were docked using the “Docking” facilities in MOE with an induced fit scheme, allowing local rearrangements of side chain atoms around docked molecules (limited by a .05kcal/mol/Å^2^ harmonic restraints on side chain atoms) in several possible binding sites. The most likely binding sites of the CCX, KZ-41, and ATP molecules in IGF-1R were identified as exhibiting the most favorable predicted binding free energies, as calculated with the GBVI/WAS dG and London dG docking scores implemented in MOE.

## Results

### Glucose-induced caspase-3 levels are reduced by KZ-41

**Fig 2:**
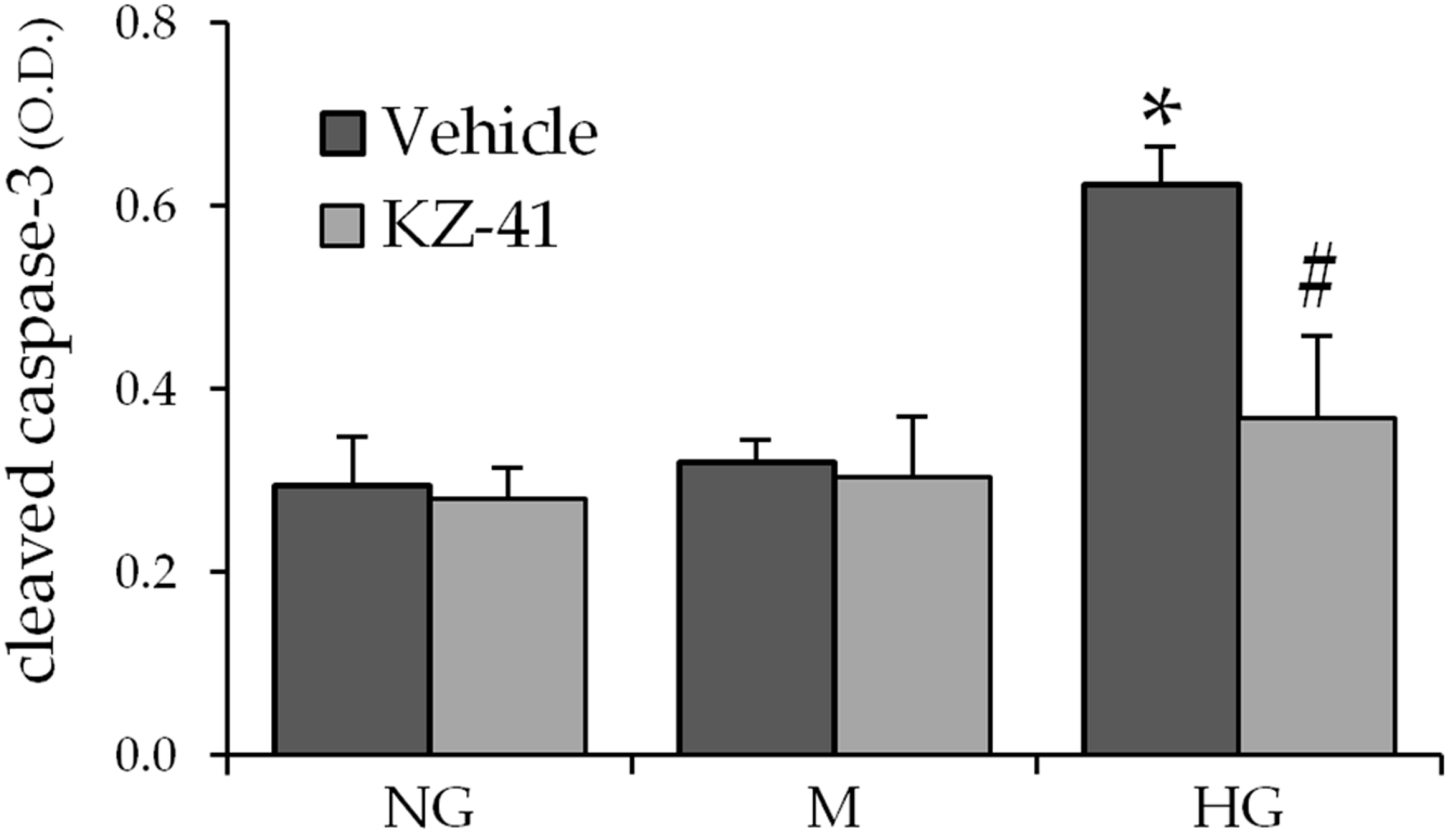
KZ-41 reverses glucose-induced caspase-3 activation. Following 24 hours serum starvation, RECs were cultured for three days in either normal glucose (5 mM, NG), high glucose (25 mM, HG), or mannitol (25 mM, M) as an osmotic control. Cells were then treated with normal saline (vehicle control) or KZ-41 (10 μM) for two hours. Cleaved caspase-3 levels in REC lysates were measured using the PathScan ELISA assay. Cleaved caspase-3 levels were significantly elevated after prolonged HG exposure. KZ-41 decreased HG-induced cleaved caspase-3 levels. Data are presented as optical density (O.D.) at 450 nm and represent mean ± S.D. (n = 6). *P < 0.05 versus NG or M (normal saline control and KZ-41 treated), #P < 0.05 versus HG control.

Apoptotic cell death is triggered in RECs continuously exposed to high glucose concentrations [3]. Activated (cleaved) caspase-3, a crucial effector of the terminal or execution phase of the apoptotic pathway, has been recognized as a reliable phenotypic marker of apoptosis [22]. Thus, to determine the effect of KZ-41 on glucose-induced apoptosis, cultured REC cells were exposed to HG, NG, or M (as an osmotic negative control) and either KZ-41 or normal saline (vehicle). Similar to previous reports [23], cleaved caspase-3 (Asp 175) levels in RECs exposed to HG were significantly higher when compared to RECs cultured in either NG or M (Fig 2). KZ-41 significantly reduced cleaved caspase-3 levels in RECs exposed to HG (Fig 2). Whereas, cleaved caspase-3 levels were unaltered by KZ-41 in RECs cultured in either NG or M. Together, these results indicate that KZ-41 reverses glucose-induced caspase-3 activation without affecting constitutive caspase-3 levels in RECs.

### KZ-41 activates PI3K/Akt signaling

**Fig 3:**
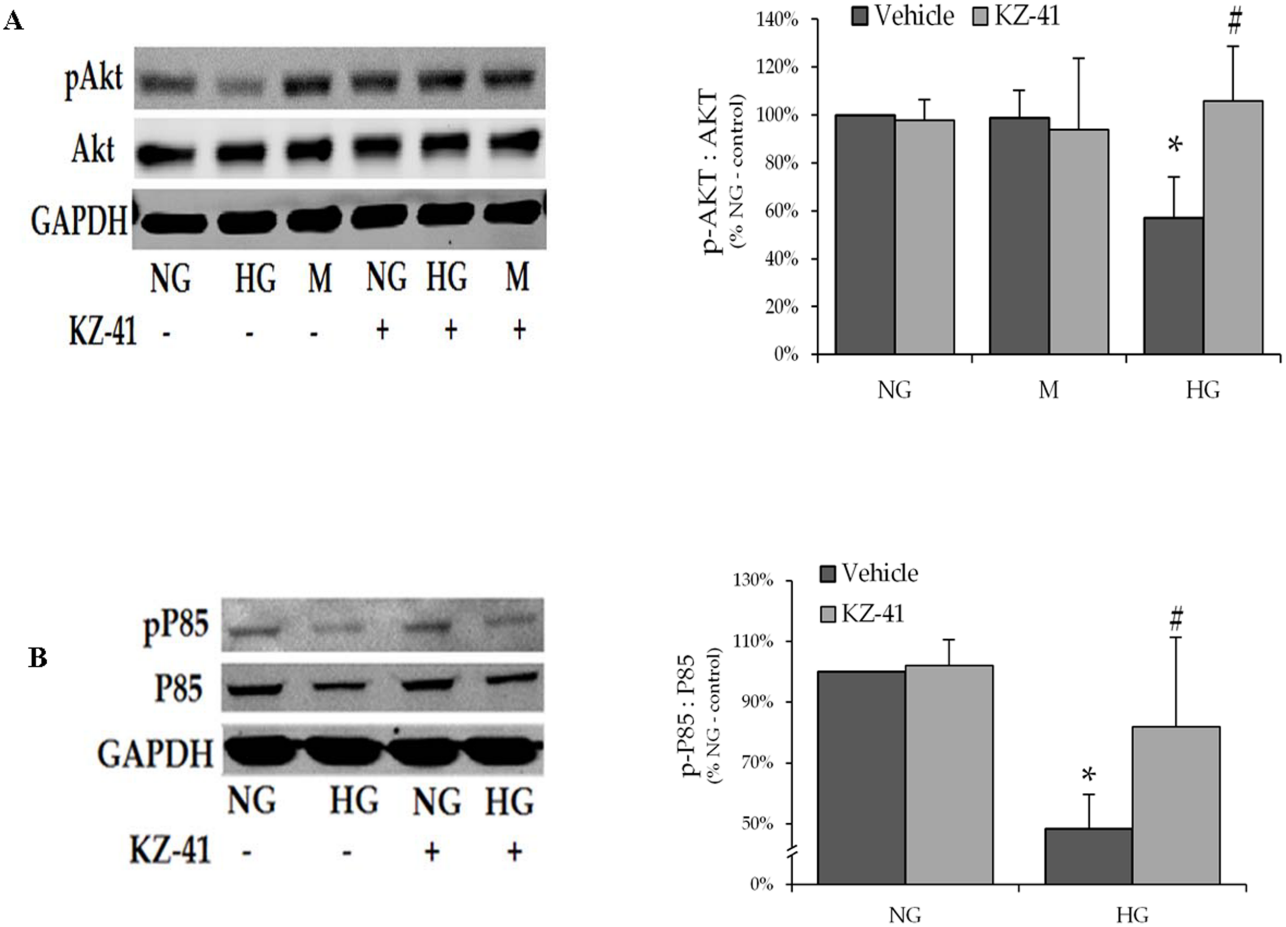
KZ-41 activates PI3K/Akt signaling. Following 24 hours serum starvation, RECs were cultured for three days in either normal glucose (5 mM, NG) or high glucose (25 mM, HG). Cells were then treated with normal saline (vehicle control) or KZ-41 (10 μM) for two hours. Cell lysates were collected for Western blot analysis using antibodies described in the Materials and methods section. Blots were probed for GAPDH as a loading control. (A) HG decreased phosphorylated (Ser473) Akt (pAkt) expression without affecting total Akt expression. KZ-41 reversed HG-induced changes in pAkt expression. (B) HG decreased phosphorylated (Tyr458) P85 (pP85) expression without affecting total P85 expression, which resulted in a reduced ratio of pP85 to P85 expression. KZ-41 enhanced pP85 expression without altering P85 expression under HG conditions. Data represent mean ± SD from three replicate experiments. *P < 0.05 versus NG or M (normal saline control and KZ-41 treated), #P < 0.05 versus HG control.

Caspase-3 activation correlates with reduced Akt activation in endothelial cells continuously exposed to high glucose [3]. Thus, we hypothesized that KZ-41 decreased caspase-3 activity by enhancing Akt expression and/or activation. To test this hypothesis, we measured the effect of KZ-41 on total and phosphorylated (Ser473) Akt levels in lysates from RECs cultured in HG. We found that HG significantly reduced Akt phosphorylation (activation) without altering total Akt expression (Fig 3A). However, KZ-41 reversed the effect of HG on phosphorylated Akt expression without changing total Akt levels. The net effect of KZ-41 treatment was restoration of the ratio of phosphorylated to total Akt expression found in RECs cultured in NG (Fig 3A).

PI3K class I_A_, which comprises an 85 kDa regulatory subunit (p85) and a 110 kDa catalytic subunit (p110), promotes pro-survival signal transduction by phosphorylating Akt [24]. To determine whether or not KZ-41 altered PI3K activity under HG conditions, we measured total and phosphorylated (Tyr458) p85 levels and found that glucose reduced both total and phosphorylated levels such that the ratio of phosphorylated to total p85 was significantly reduced compared to NG (Fig 3B). KZ-41 promoted p85 activation without altering total p85. Together, these data indicate that KZ-41 enhances PI3K/Akt signaling in RECs continuously exposed to HG for 72 hours through a mechanism involving increased activation of p85.

### Reduction in IRS-1 levels is associated with increased ERK activation

**Fig 4:**
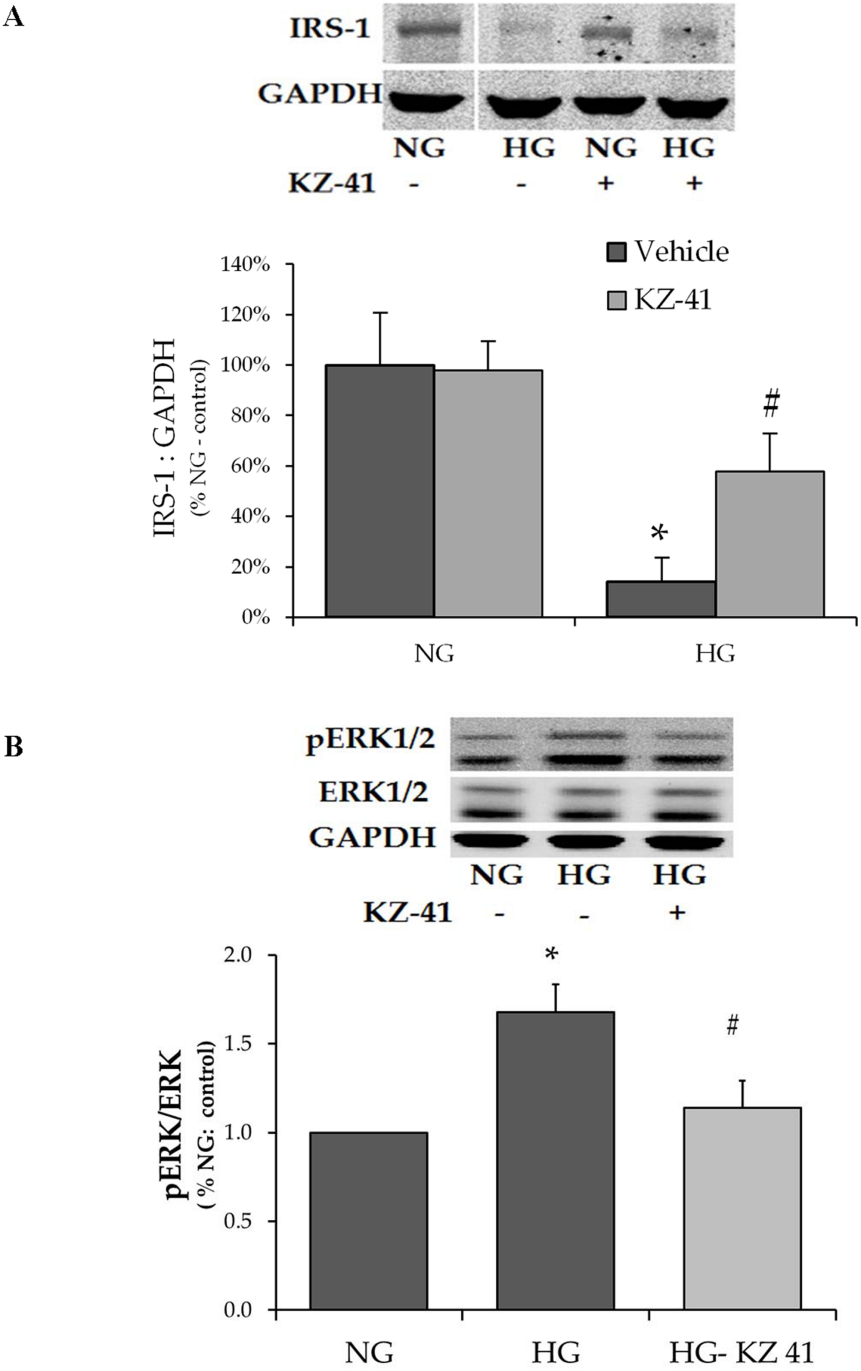
IRS-1 levels negatively correlate with ERK activation. Following 24 hours serum starvation, RECs were cultured for three days in either normal glucose (5 mM, NG) or high glucose (25 mM, HG). Cells were then treated with normal saline (vehicle control) or KZ-41 (10 μM). Cell lysates were collected at two hours or 5 minutes for Western blot analysis of IRS-1 and ERK (p42/44) expression, respectively, using antibodies described in the Materials and methods section. Blots were probed for GAPDH as a loading control. (A) IRS-1 expression was reduced in RECs cultured in HG. KZ-41 increased IRS-1 expression in RECs cultured in HG. (B) The ratio of phosphorylated (Thr202/Tyr204) to total ERK (pERK1/2:ERK1/2) was significantly increased in RECs cultured in HG. KZ-41 treatment decreased the pERK1/2:ERK1/2 ratio. Data represent mean ± SD from three replicate experiments. *P < 0.05 versus NG (normal saline control and KZ-41 treated), #P < 0.05 versus HG control.

Interaction of the Src homology 2 (SH2) domain of p85 with the docking protein IRS-1 leads to enhanced PI3K I_A_ signaling [25]. To determine IRS-1’s involvement in glucose-induced changes in PI3K/Akt signaling, we measured IRS-1 levels in RECs cultured in high glucose for 72 hours (Fig 4A). High glucose dramatically decreased IRS-1 total protein levels; an effect that was significantly reversed by KZ-41. IRS-1 degradation rate is controlled by phosphorylation at critical serine and tyrosine sites. For example, ERK negatively regulates IGF-1-dependent PI3K signaling by targeting IRS-1 for serine phosphorylation (636/639) and degradation [26]. Our results indicate that phosphorylated ERK levels are significantly increased by prolonged high glucose exposure and that KZ-41 inhibits glucose-induced ERK activation (Fig 4B). Together, these data suggest that glucose-induced ERK activation contributes to reduced IRS-1 levels. Moreover, KZ-41’s inhibition of ERK activation provides a potential mechanistic basis for increased IRS-1 protein levels.

### HG represses IGF-1R phosphorylation (activation)

**Fig 5:**
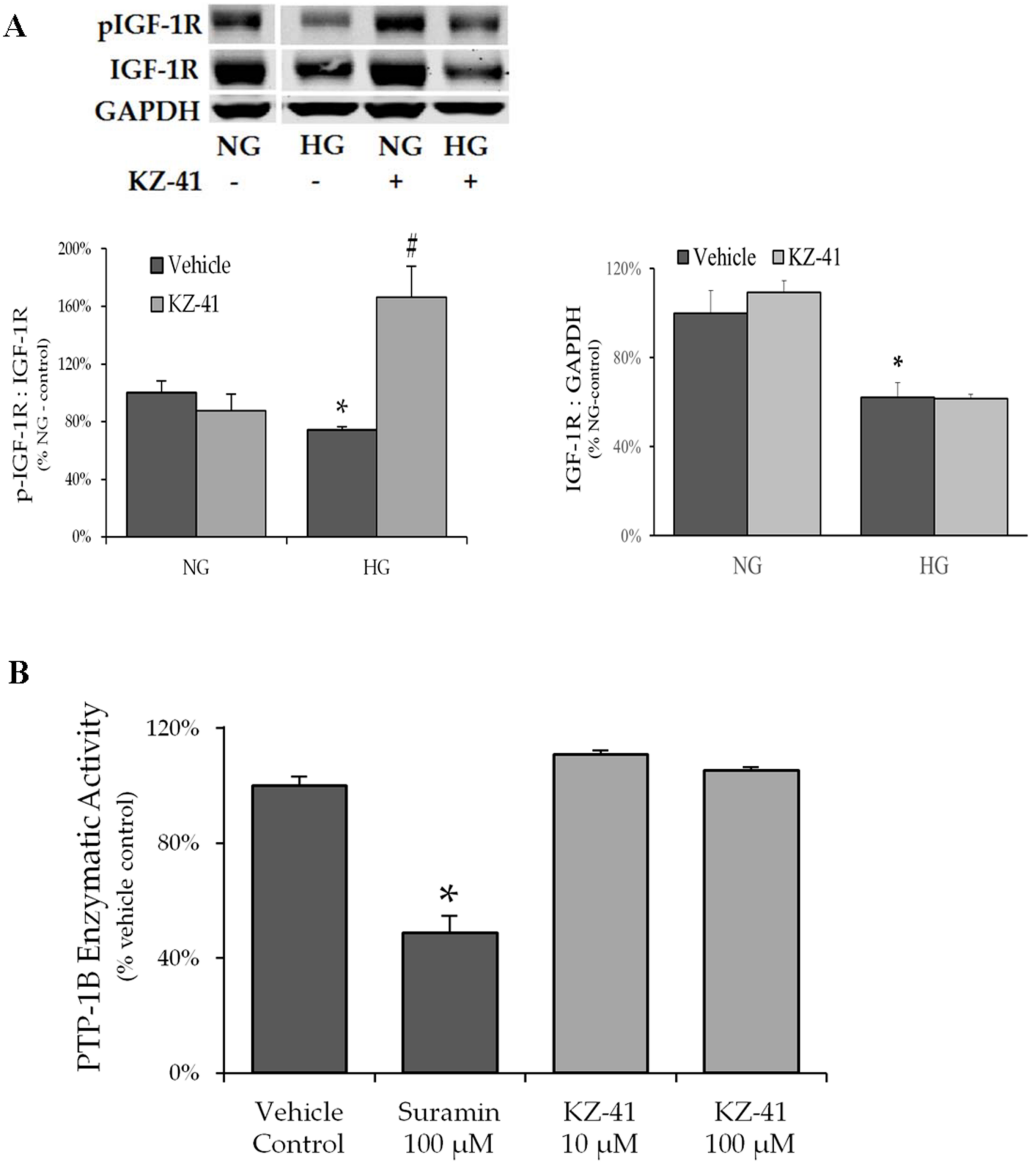
KZ-41 triggers IGF-1R autophosphorylation. Following 24 hours serum starvation, RECs were cultured for three days in either normal glucose (5 mM, NG) or high glucose (25 mM, HG). Cells were then treated with normal saline (vehicle control) or KZ-41 (10 μM) for two hours. Cell lysates were collected for Western blot analysis using antibodies described in the Materials and methods section. Blots were probed for GAPDH as a loading control. PTP1B activity was measured in a cell-free system using a colorimetric purified recombinant enzyme assay. PTP1B enzyme, IR5 phosphate substrate, and either KZ-41 (10 and 100 μM) or suramin (100 μM), a PTP1B inhibitor, were incubated 96-well microtiter plate for one hour at 30°C. Color was allowed to develop for 30 minutes and UV absorbance was measured at 620 ηm using a microplate reader. (A) IGF-1R activation was lessened by HG as evidenced by reduction in both total and phosphorlyated (Tyrosine1135/1136) IGF-1R expression. Under HG conditions, KZ-41 treatment enhanced IGF-1R phosphorylation without impacting total IGF-1R expression. *P < 0.05 versus NG (normal saline control and KZ-41 treated), #P < 0.05 versus HG control. (B) PTP1B activity was inhibited by suramin, whereas KZ-41 failed to inhibit PTP1B enzymatic activity when tested up to 100 μM. *P < 0.05 versus vehicle control. Data represent mean ± SD from three replicate experiments.

Autophosphorylation of the insulin receptor, as well as the highly homologous IGF-1R, leads to tyrosine phosphorylation of both IRS-1 and IRS-2 and activation of PI3K I_A_ activity [25]. IGF-1R and IGF-1 protein expression are altered in diabetic human REC cultures suggesting IGF-1 signaling is impaired [27]. Thus, we measured total and phosphorylated (Tyr1135/Tyr1136) IGF-1R levels in RECs to determine if glucose-induced changes in IGF-1R activation contributed to reduced IRS-1 levels and PI3K/Akt signaling (Fig 5A). We found that high glucose inhibited total and phosphorylated IGF-1R levels with the net result being a significant reduction in the ratio of phosphorylated to total IGF-1R (Fig 5A). KZ-41 dramatically increased IGF-1R phosphorylation without altering total IGF-1R (Fig 5A).

One potential explanation for these data is that KZ-41 enhances IGF-1R phosphorylation by inhibiting phosphatase activity. To test this hypothesis, we measured KZ-41’s effect on the non-transmembrane phosphatase protein-tyrosine phosphatase 1B (PTP1B), which regulates IGF-1 signaling by inhibiting IGF-I-induced receptor autophosphorylation and IRS protein phosphorylation [28]. Our results indicate that suramin, a PTP1B inhibitor [29], inhibited PTP1B activity, whereas KZ-41 failed to inhibit phosphatase activity even at concentrations up to 100 μM (Fig 5B). Together, these data support the notion that KZ-41 induces IGF-1R autophosphorylation through a PTP1B-independent mechanism.

### KZ-41’s pro-survival mechanism of action requires the IGF-1R

**Fig 6:**
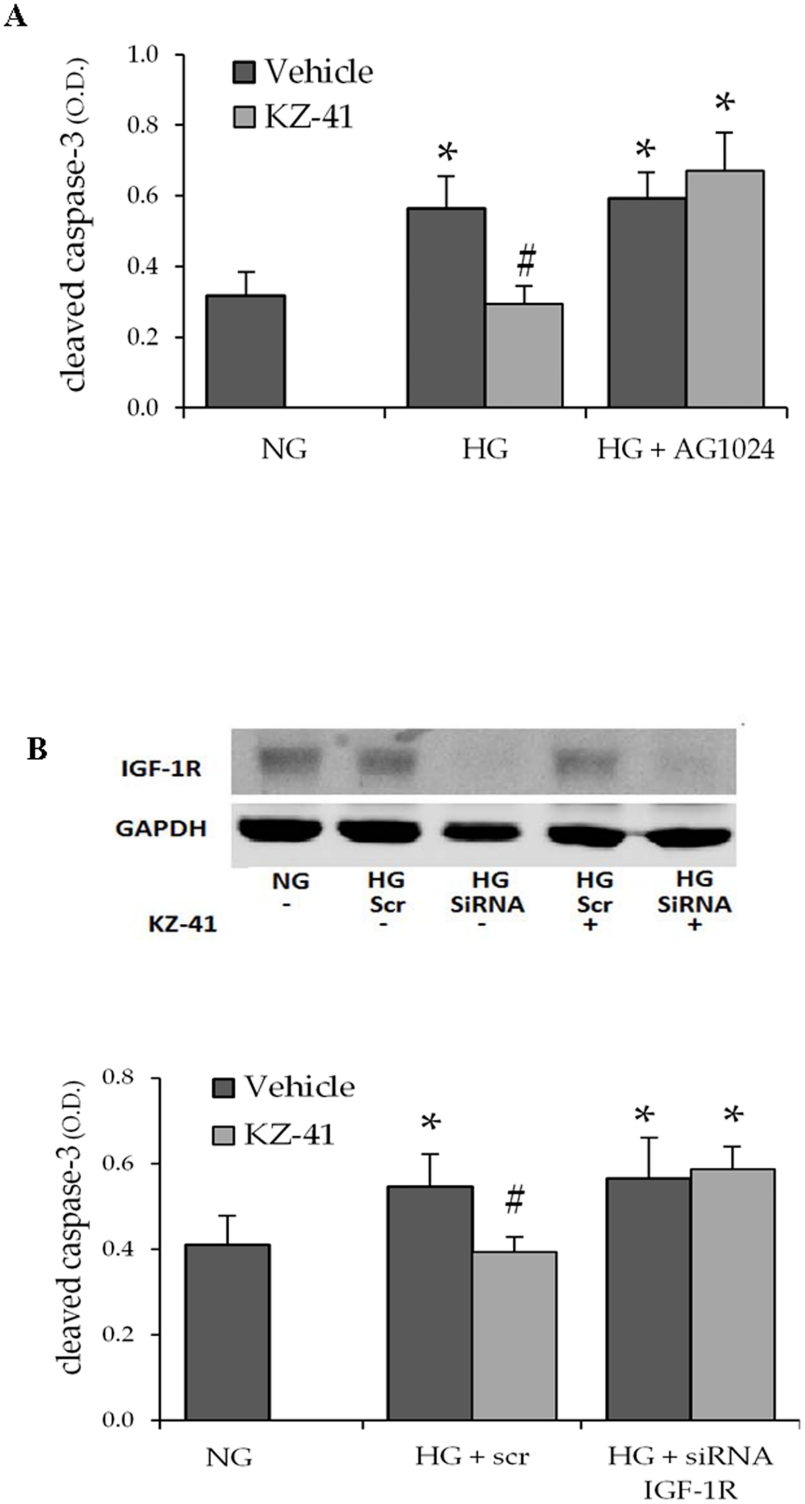
IGF-1R is required for KZ-41’s pro-survival mechanism. (A) Following 24 hours serum starvation, RECs were cultured for three days in either normal glucose (5 mM, NG) or high glucose (25 mM, HG). Cells were then treated with normal saline (vehicle control) or KZ-41 (10 μM) for two hours. In a separate cohort, RECs were treated with the IGF-1R tyrosine kinase inhibitor AG1024 (10 μM) for two hours prior to the addition of KZ-41. Cleaved caspase-3 levels were measured as previously described. As before, KZ-41 rescued RECs from death following HG exposure. However, KZ-41 was unable to reduce HG-induced cleaved caspase-3 levels following pre-treatment with AG1024. Data are presented as optical density (O.D.) at 450 nm and represent mean ± S.D. (n = 6). (B) RECs transfected with scrambled (control) or IGF-1R siRNA were cultured as described above. Cell lysates were collected for Western blot analysis using antibodies described in the Materials and methods section. Blots were probed for GAPDH as a loading control. KZ-41 reduced cleaved caspase-3 levels in mock transfected RECs cultured in HG. However, cleaved caspase-3 levels were unaffected by KZ-41 in IGF-1R siRNA RECs. Data represent mean ± SD from three replicate experiments. *P < 0.05 versus NG (normal saline control), #P < 0.05 versus HG control.

IGF-1R kinase impaired mutant cells fail to activate PI3K/Akt suggesting autophosphorylation is required for IGF-1’s pro-survival effect [30]. We used pharmacologic and genomic knockdown approaches to determine the reliance of KZ-41’s pro-survival effect on the IGF-1R. First, using a selective inhibitor of IGF-1R autophosphorylation, AG1024 [31], we tested the hypothesis that phosphorylation is required for the pro-survival mechanism of action of KZ-41 in RECs exposed to high glucose (Fig 6A). We found that blockade of IGF-1R kinase activity completely reversed the effect of KZ-41 on glucose-induced cleaved caspase-3 levels. Next, we silenced IGF-1R expression to confirm the reliance of KZ-41’s mechanism of action on the IGF-1R (Fig 6B). IGF-1R knockdown, verified by Western blot, was successful in RECs transfected with siRNA, but not in RECs transfected with scrambled (control) RNA. IGF-1R silencing blunted KZ-41’s ability to reduce caspase-3 activation under high glucose conditions (Fig 6B). Together these data suggest that autophosphorylation of the IGF-1R is critical to the pro-survival mechanism of action of KZ-41 in RECs exposed to high glucose.

### Computational docking suggests that KZ-41may bind in multiple possible sites

In order to identify and characterize possible mechanisms by which KZ-41 activated the IGF-1R, molecular modeling approaches were used as described in Methods to suggest possible binding modes between KZ-41 and the kinase domain of the IGF-1R. As described in Methods, three different homology models of the IGF-1R kinase domain were used to perform a virtual docking of CCX, KZ-41, and ATP. The backbone structures of the different models exhibit relatively minor differences in the modeled regions. All the residues of all the homology models in these modeled regions, but for one of them (Leu 1104), are in allowed regions of the Ramachandran plot (S1 Fig). This (small scale) “ensemble docking” approach allows the protein target to sample a conformational space beyond that of the constrained crystal structure, and essentially aims at allowing (albeit limited in the present study) a conformational selection mechanism for binding of ligands [32].

**Fig 7:**
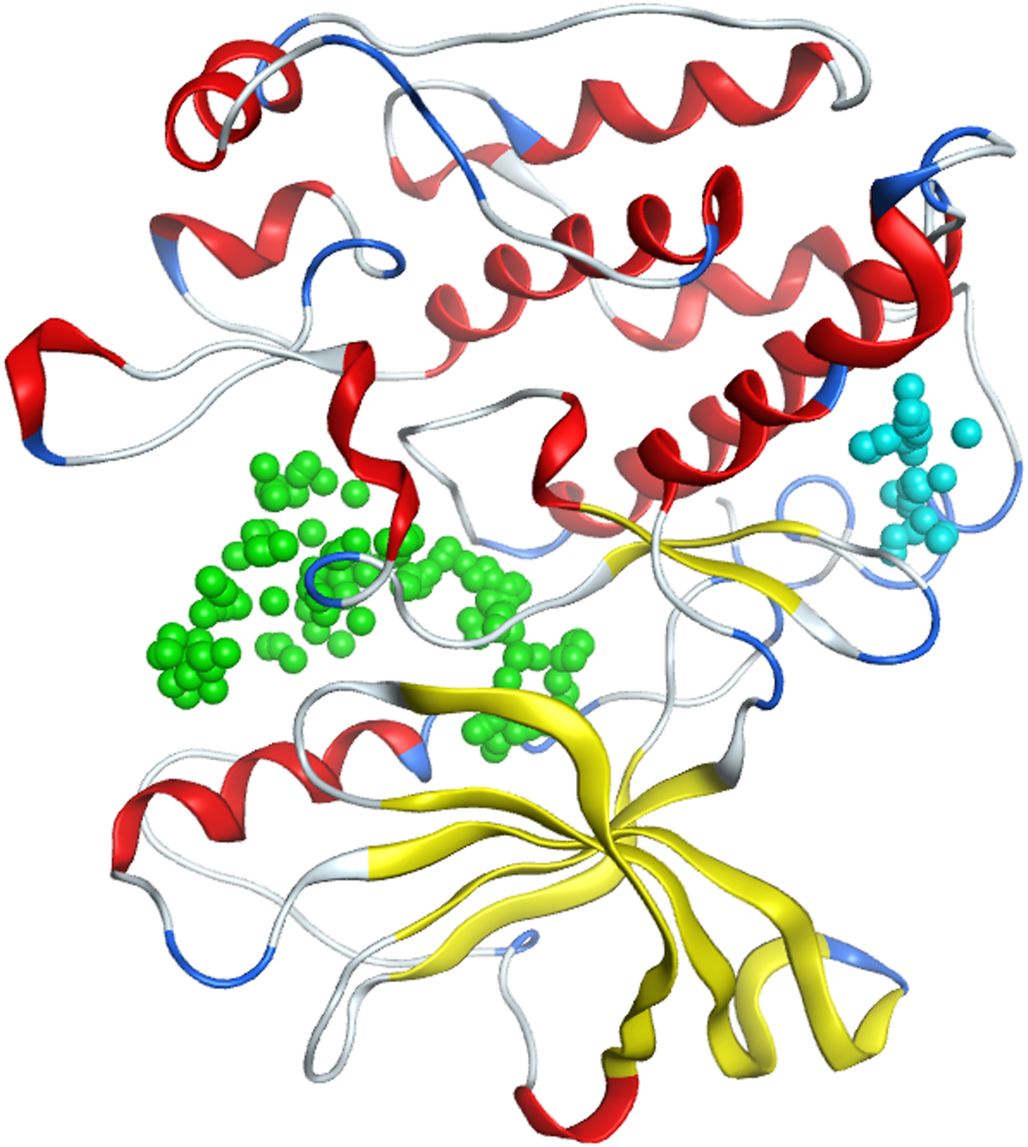
IGF-1R homology model. Site 1 (kinase site) is in green, and Site 2 (potential allosteric site) is in blue.

**Fig 8:**
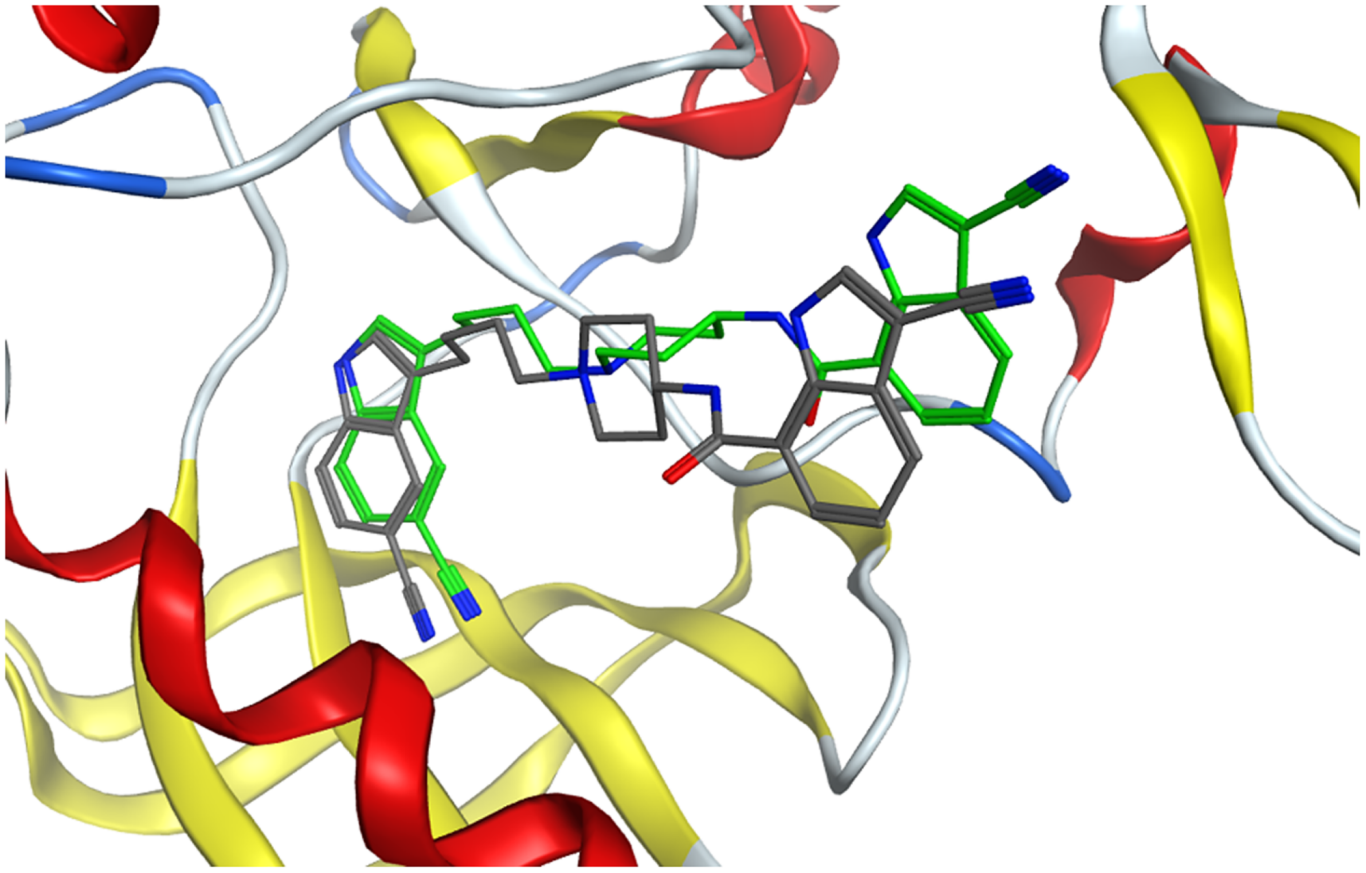
Binding mode of CCX in the kinase Site 1 domain. Green carbon atoms: CCK co-crystallized form the PDB structure. CPK colors: CCX docked using MOE in the protein models.

In each of these three models, two possible binding sites were identified (Fig 7). Site 1, the kinase site, corresponds to the CCX binding site in the crystal structure. Site 2, a potential allosteric site, is located posterior to the hinge region and contains two CCX molecules that bridge two different IGF-1R monomers together in the unit cell of the crystal structure. CCX was computationally docked in Site 1, and its position is similar to that in the crystal structure, confirming that docking approaches in MOE can correctly identify binding modes of small molecules in the protein (Fig 8 and S2 Fig) with a 0.9 Å RMSD between the docked and co-crystalized ligand structures. KZ-41 was docked in Site 1 and Site 2, and ATP was docked alone as well as in a mixture with KZ-41.

**Fig 9:**
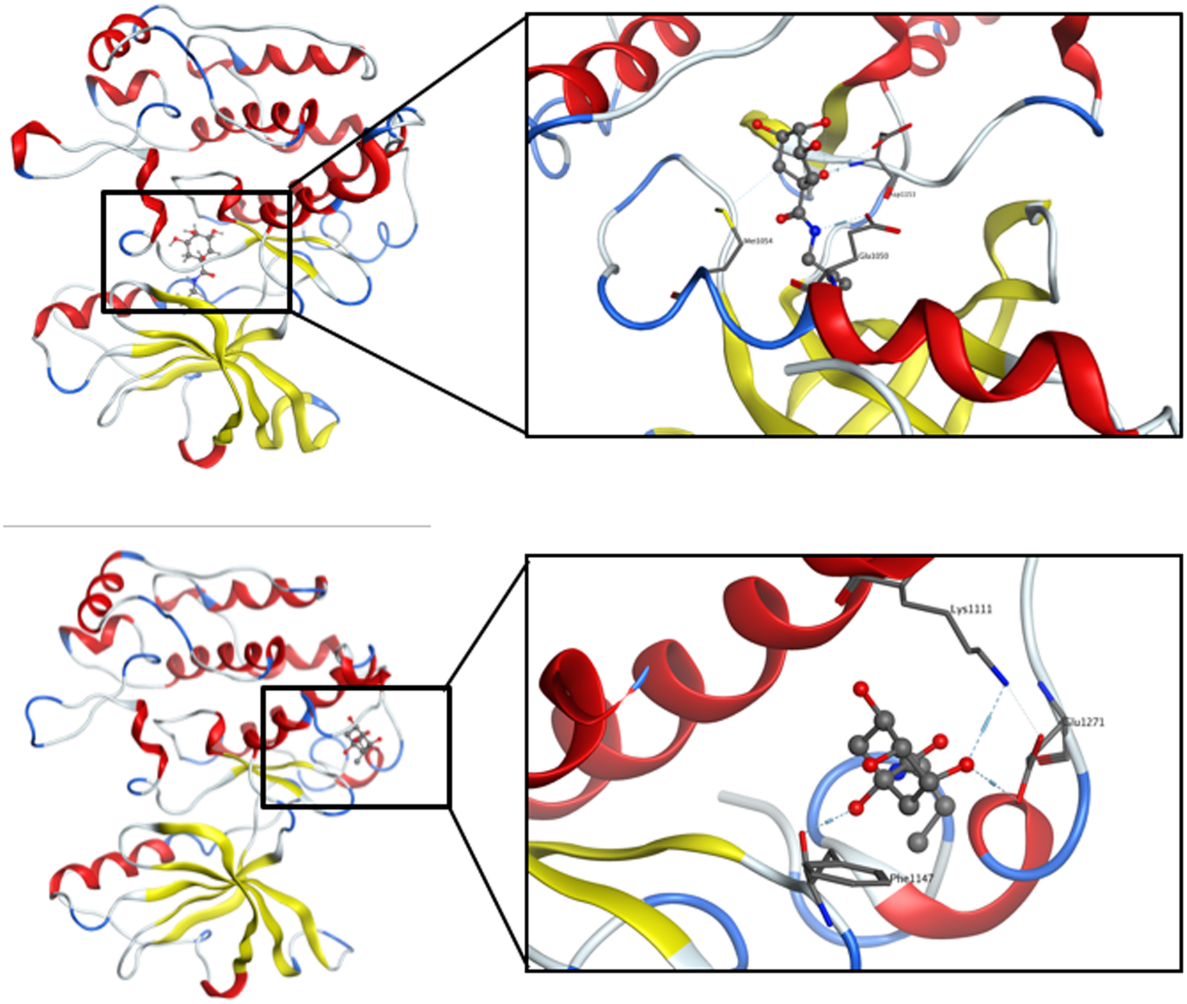
Optimal binding modes of KZ-41 determined by scoring functions. (A) Best binding mode of KZ-41 as predicted by the GBI/WSA scoring function, i.e., in Site 1. Left: global view, right: zoomed in on binding region. (B) Best binding mode of KZ-41 as predicted by the London dG scoring function, i.e., in Site 2. Left: global view, right: zoomed in on binding region.

**Fig 10:**
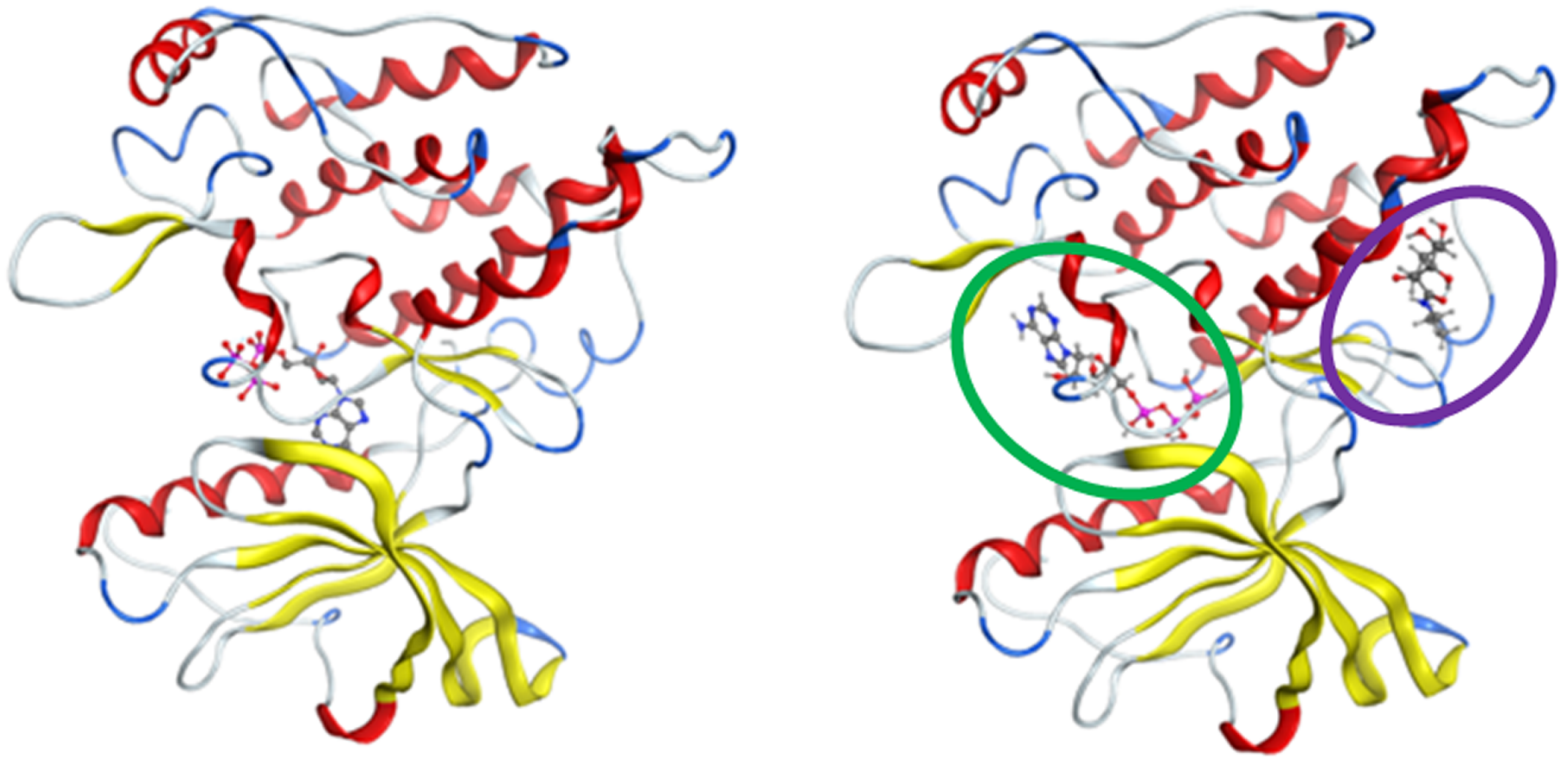
Optimal binding mode of ATP determined by scoring functions. (A) Best predicted binding mode of ATP. (B) Best predicted binding modes of ATP in the presence of KZ-41. ATP is predicted to bind in Site 1 (green oval) and KZ-41 is predicted to bind in site 2 (purple oval).

The most favorable predicted binding scores of CCX, KZ-41, and ATP in each of the three snapshots and each of the two binding sites are given in Table 1 for both scoring functions described in Methods, and the corresponding binding modes are shown in Figs 9 and 10. While CCX and ATP are predicted to bind unequivocally in the kinase domain Site 1, KZ-41 binding is ambiguous. One of the two scoring functions used in our calculations, GBVI/WSA, ranks binding of KZ-41 in Site 1 as best, while the other scoring function, London dG, ranks binding of KZ-41 in the potentially allosteric site, Site 2, as best. While co-binding of ATP and KZ-41 together in Site 1 was investigated, the calculation results suggest that binding of these two molecules could happen in different protein sites. The tyrosine domain responsible for autophosphorylation is located on the A-loop of the structure, which is shown to be close to the predicted bound ATP in Fig 10, strengthening the prediction of ATP binding location.

**Table 1:** Docking scores.

Molecule/Site	GBVI/WSA	GBVI/WSA dG Normalized	London dG	London dG Normalized
**CCX**	-9.7	-0.28	-15.5	-0.44
**KZ-41 (Site 1)**	-6.9	-0.43		
**KZ-41 (Site 2)**			-11.2	-0.70
**ATP (Site 1)**	-9.8	-0.31	-15.6	-0.50
**ATP (Site1) with KZ-41 (Site2)**	-9.3	-0.29	-14.1	-0.46

Docking scores as calculated in MOE for GBVI/WSA and London scoring functions for the best binding modes of CCX, KZ-41 and ATP in any of the three homology models. All energies are in kcal/mol. “Normalized” scores are docking scores divided by the number of heavy atoms in each molecule.

## Discussion

**Fig 11:**
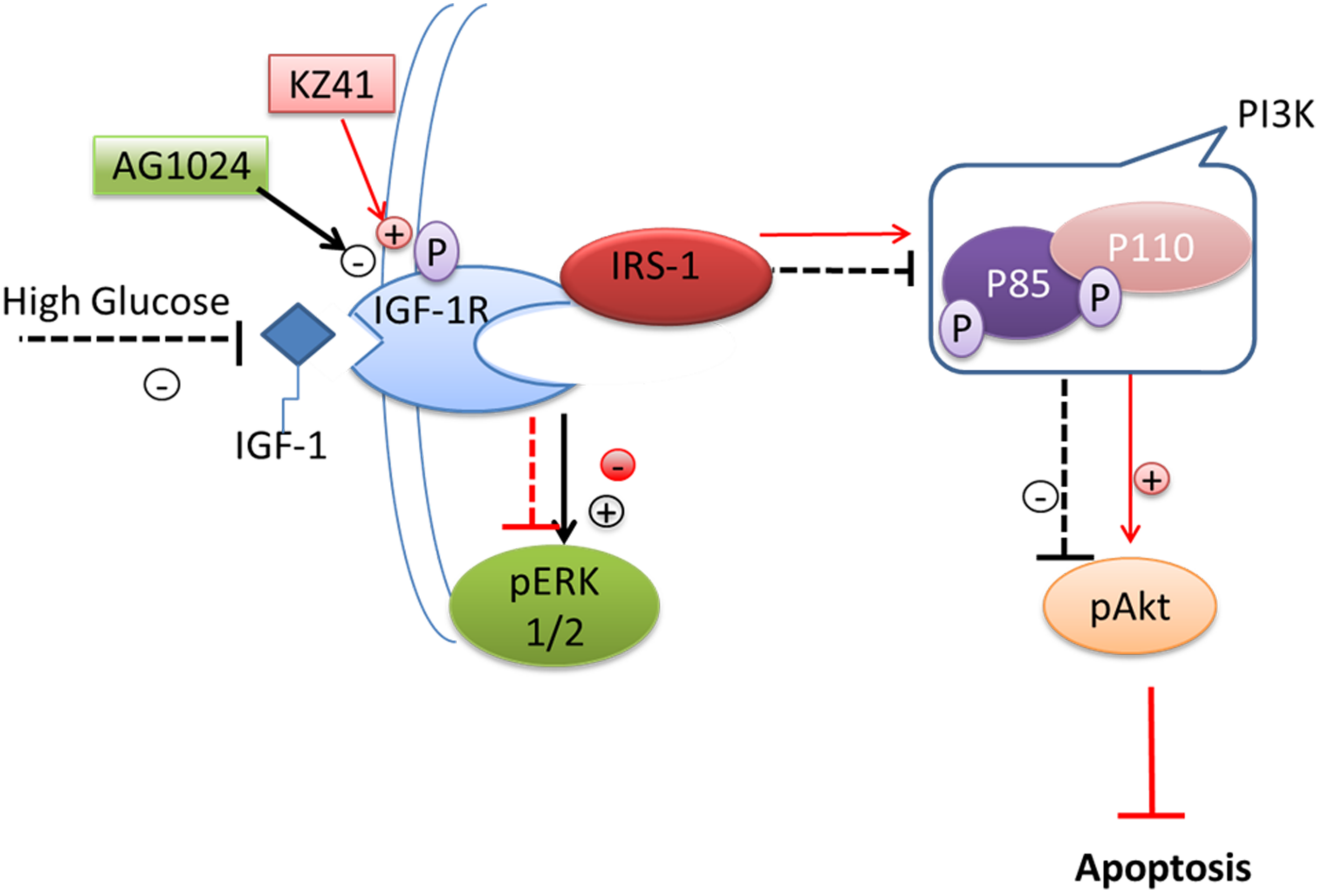
Proposed model describing activation of IGF-1R-dependent pro-survival signal transduction by KZ-41. HG reduces IGF-1R-dependent Akt pro-survival signaling and promotes REC migration via ERK up-regulation. KZ-41 activates the IGF-1R, while simultaneously inhibiting glucose-induced ERK activation. Consequently, KZ-41 protects RECs from glucose-induced apoptosis.

There is an active debate in the literature as to whether or not DR begins with abnormalities in the neuronal and glial cells of the retina or in the retinal blood vessels. Regardless of the initiating event(s), it is clear that microaneurysms in the retina represent the first clinically observable manifestation of disease [1]. Microaneurysms are associated with REC and pericyte loss, which leads to capillary dropout and development of retinal ischemia. Thus, protection of capillary cellular components has been the focus of intensive research in identifying novel targets for treatment of both non-proliferative (NPDR) and proliferative DR (PDR). In the present study, we demonstrate that a quinic acid derivative, KZ-41, rescues RECs from high glucose-induced apoptosis by enhancing pro-survival signaling through PI3K/Akt. Pharmacologic and genomic knockdown experiments identify the IGF-1R as indispensable to KZ-41’s pro-survival mechanism of action (Fig 11).

Upon IGF-1 binding, the activation loop located in the IGF-1R kinase (IGF-1RK) domain rotates to reveal an ATP binding site. Simultaneously, the two kinase domains of the dimeric IGF-1R are brought into close proximity leading to catalysis and *trans* phosphorylation of three conserved tyrosine residues (Tyr 1131, 1135, and 1136) located on the activation loop [33]. Our finding that KZ-41 elicits Tyr 1135/1136 phosphorylation suggests that KZ-41, directly or indirectly, triggers re-positioning of the activation loop. Direct activation of tyrosine kinase receptors by small molecules has precedent. For example, the glucose analog, 6Cl-TGQ, was found to bind the IR and trigger IR autophosphorylation. Interestingly, despite high homology to the IR kinase domain (84%), 6Cl-TGQ was unable to initiate IGF-1R autophosphorylation [34]. IGF-1R transactivation through indirect mechanisms has also been described [35–37]. For example, aldosterone transactivates the IGF-1R in a mineralocorticoid/glucocorticoid receptor-dependent manner [36]. The ATP docking scores alone and that with KZ-41 are similar, suggesting a possible indirect activation mechanism that bears further investigation via molecular dynamics and additional docking simulations.

R- cells, a 3T3-like mouse fibroblast with a targeted disruption of the IGF-1R, over-expressing kinase impaired human IGF-1R fail to activate Akt in response to IGF-1 stimulation [30]. However, IGF-1-dependent ERK activation was unaffected suggesting ERK signaling is independent of IGF-1R autophosphorylation. So, how does IGF-1 activate ERK signal transduction? Recent studies demonstrate that IGF-1 binding leads to IGF-1R phosphorylation by the G protein-coupled receptor (GPCR) kinase-6 (GRK6), β-arrestin-1 recruitment, receptor internalization, and ERK activation [38]. Our finding that KZ-41, unlike IGF-1, inhibits ERK activity raises the interesting possibility that KZ-41 selectively activates Akt signaling under high glucose stress.

The concept of biased agonism, a phenomenon well-described for GPCRs (reviewed in [39]), has recently been demonstrated for the IGF-1R [40]. The cathelicidin peptide LL-37, an IGF-1R agonist, selectively activates ERK leading to enhanced cell migration and invasion of MCF-7 human breast cancer cells. IGF-1 protects RECs from high glucose-induced apoptosis through the PI3K/Akt pathway, whereas proliferation requires ERK activation [12]. In fact, it is surmised that IGF-1 anti-apoptotic effects are predominantly driven by the activation of PI3K/Akt signaling, independent of ERK activation—as sustained levels of ERK-mediated signaling has been positively correlated with numerous pro-apoptotic signaling pathways in response to oxidative stress, *in vivo* [41, 42]. Thus, identification of therapeutics that bias the IGF-1R to selectively activate Akt would address potential concerns that IGF-1 may exacerbate microvascular dysfunction in diabetes through ERK, as it can also play a role in VEGF-induced RNV [43] since IGF-1-induced VEGF expression is ERK-dependent [44]. Biasing the IGF-1R away from ERK-activation could also have important implications in preventing ERK-dependent migration and invasion of highly malignant cancer cells [40].

In light of these data, we have built a rationale for targeting the IGF-1R with KZ-41 to bias the receptor’s activation pathway away from ERK and towards the PI3K/Akt signaling arc. We explored the binding mechanisms of KZ-41 within the kinase domain and hinge-region of the IGF-1R using a docking approach. We investigated the IGF-1R kinase domain containing a co-crystallized IGF-1R inhibitor, CCX [20, 21], for potential KZ-41 binding sites. The computational modeling presented here suggests two potential KZ-41 binding sites within the IGF-1R kinase structure; both sites are capable of binding KZ-41. The calculated binding scores are too close to each other, and too dependent on a specific scoring function, to assertively suggest that one site is preferential to the other.

Two intriguing possibilities arise from our modeling results. First, KZ-41 can indeed bind to IGF-1RK, and that, within the accuracy of the modeling calculations, it could bind at either the allosteric site or the “main” site. Binding at either site also affects the docking scores and positioning of ATP. The position of ATP in each case has the potential to interact with the tyrosine residues responsible for autophosphorylation. The residue Tyr1135, which is the first of the three tyrosine residues involved in phosphorylation [45], is positioned close to ATP in the present docking calculations. The positioning of ATP also directly competes with CCX, and therefore concentration as well as free energy may affect whether ATP or CCX binds in the kinase S1 site.

The mechanism(s) through which KZ-41 exhibits its agonist properties is not clearly suggested by the calculations. It is interesting to note, however, that KZ-41 is predicted to potentially bind preferably in Site 2 around which the opening of the active site takes place (15), albeit with a lower binding affinity for the protein than the CCX inhibitor. It is possible, although at this point it is only a hypothesis, that KZ-41 can bind and stabilize a conformation of the protein that would favor binding of ATP and hinder binding of CCX. Future molecular dynamics simulations will provide additional structural snapshots to be used in ensemble docking calculations to correlate the dynamic variations of the protein and the conformations selected by different ligands, agonists and antagonists. This will be important in further differentiating between binding site preferences of KZ-41 within these locations.

In conclusion, this study provides the first description of a detailed pro-survival mechanism of action, including potential target identification, for QA and its analogs. Specifically, we demonstrate that a QA derivative, KZ-41, functions as a survival factor for RECs by activating IGF-1R/IRS-1/PI3K/Akt signal transduction and inhibiting caspase-3 activation. Moreover, the divergent effects of KZ-41 on Akt and ERK signaling support previous studies that demonstrate the feasibility of functional separation of IGF-1R-dependent downstream Akt and ERK signal transduction. These data, together with previous proof-of-concept efficacy studies demonstrating KZ-41 mitigates pathologic retinal neovascularization in the murine OIR model, suggest QA derivatives may offer therapeutic benefit in DR. On-going studies are focused on identifying whether QA analogs activate the IGF-1R via direct binding or through transactivation, as well as, characterizing the pharmacologic effect of QA analogs on neuronal and vascular changes in the diabetic rat retina.

## Supporting information

S1 Fig Similarities and differences amongst IGF1-R models. Left: superposition of the homology models of the protein target. Each model has a different backbone color (blue, green, red). Right: Ramachandran map of the residues in the modeled regions of the protein target (in red ovals on the Left).
(TIF)

S2 Fig Docking of CCX compared to co-crystalized CCX in IGF-1R structure. Superposition of the co-crystalized ligand (green) and the re-docked ligand (yellow) using the London dG scoring function in the protein structure. Left: whole-protein view, right: zoom-in on the binding site.
(TIF)
