# Supplementary figures and images for: The quinic acid derivative KZ-41 prevents glucose-induced caspase-3 activation in retinal endothelial cells through an IGF-1 receptor dependent mechanism
Source: PLoS One. 2017 Aug 10;12(8):e0180808. doi: 10.1371/journal.pone.0180808 (PMC5552119; doi:10.1371/journal.pone.0180808)

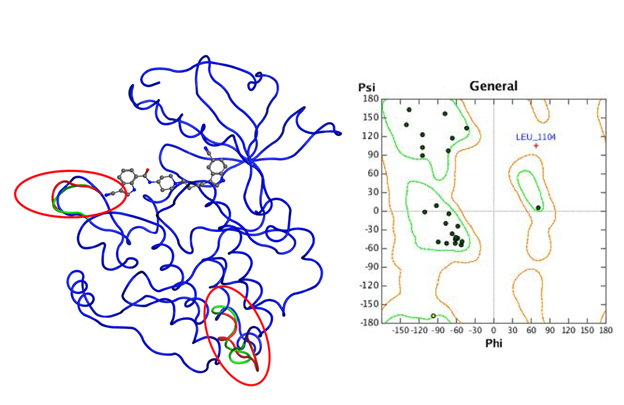

Supplement: S1 Fig — Left: superposition of the homology models of the protein target. Each model has a different backbone color (blue, green, red). Right: Ramachandran map of the residues in the modeled regions of the protein target (in red ovals on the Left). (TIF) [file pone.0180808.s001.tif]

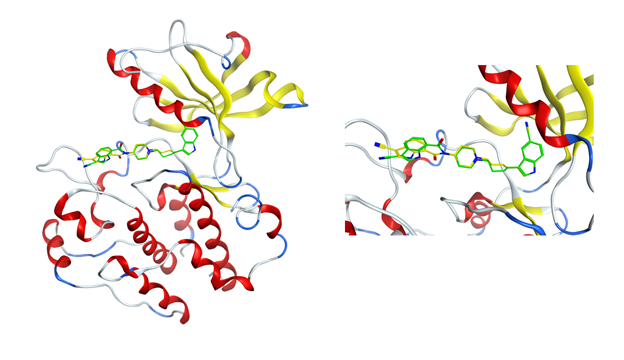

Supplement: S2 Fig — Superposition of the co-crystalized ligand (green) and the re-docked ligand (yellow) using the London dG scoring function in the protein structure. Left: whole-protein view, right: zoom-in on the binding site. (TIF) [file pone.0180808.s002.tif]
